# An Adjunctive Internet-Based Intervention to Enhance Treatment for Depression in Adults: Randomized Controlled Trial

**DOI:** 10.2196/26814

**Published:** 2021-12-16

**Authors:** J Carola Pérez, Olga Fernández, Cristián Cáceres, Álvaro E Carrasco, Markus Moessner, Stephanie Bauer, Daniel Espinosa-Duque, Sergio Gloger, Mariane Krause

**Affiliations:** 1 Facultad de Psicología Universidad del Desarrollo Santiago Chile; 2 Departamento de Psiquiatría y Salud Mental Facultad de Medicina Universidad de Chile Santiago Chile; 3 Psicomedica, Clincal & Research Group Santiago Chile; 4 Instituto Milenio para la Investigación en Depresión y Personalidad Santiago Chile; 5 Center for Psychotherapy Research University Hospital Heidelberg Germany; 6 Facultad de Psicología Universidad CES Medellín Colombia; 7 Departamento de Psiquiatría y Salud Mental, Campus Oriente Facultad de Medicina Universidad de Chile Santiago Chile; 8 Escuela de Psicología Facultad de Ciencias Sociales Pontificia Universidad Católica de Chile Santiago Chile

**Keywords:** depression, e-mental health, blended care, internet

## Abstract

**Background:**

Internet-based interventions promise to enhance the accessibility of mental health care for a greater number of people and in more remote places. Their effectiveness has been shown for the prevention and treatment of various mental disorders. However, their potential when delivered as add-on to conventional treatment (ie, blended care) is less clear.

**Objective:**

The aim of this study is to study the effectiveness of an internet intervention (ASCENSO) implemented in addition to face-to-face treatment as usual (TAU) for depression.

**Methods:**

A 2-arm, parallel-group, randomized controlled trial was conducted in an outpatient private mental health care center in Chile. In all, 167 adults, diagnosed with major depressive disorder, without severe comorbidities, and with internet access, were included. Eighty-four participants were assigned to the intervention group and received medical and psychological TAU from the mental health center plus access to the ASCENSO online platform. The control group (n=83) received only TAU. The ASCENSO platform includes psycho-educational information, depressive symptom monitoring and feedback, and managing emergencies based on the principles of cognitive behavioral therapy. Emergency management was mental health provider–assisted. TAU includes access to primary care physicians and psychiatrists, to a brief individual psychotherapy, and to medication when needed. The baseline questionnaires were administered in person, and 6- and 9-months assessments were conducted online. Depression symptoms and quality of life were measured by self-administered questionnaires, and treatment adherence was determined via the Mental Health Center’s internal records. The usage of ASCENSO was assessed by server logs. Reduction on depressive symptomatology was considered as the primary outcome of the intervention and quality of life as a secondary outcome.

**Results:**

Of the 84 participants in the intervention group, 5 participants (6%) never accessed the online platform. Of the remaining 79 participants who accessed ASCENSO, 1 (1%, 1/79) did not answer any of the symptom questionnaire, and most participants (72/79, 91%) answered the monitoring questionnaires irregularly. The ASCENSO intervention implemented in addition to face-to-face care did not improve the outcome of the usual care delivered at the mental health center, either in terms of reduction of depressive symptoms (*F*_2,6087_= 0.48; P=.62) or in the improvement of quality of life (EQ-5D-3L: *F*_2,7678_=0.24; P=.79 and EQ-VAS: *F*_2,6670_= 0.13; P=.88). In contrast, for the primary (*F*_2,850_=78.25; P<.001) and secondary outcomes (EQ-5D-3L: *F*_2,1067_=37.87; EQ-VAS: *F*_2,4390_= 51.69; P<.001) in both groups, there was an improvement from baseline to 6 months (P<.001), but there was no change at 9 months. In addition, no effects on adherence to or use of TAU were found. Finally, the dropout rate for the face-to-face treatment component was 54% (45/84) for the intervention group versus 39% (32/83) for the control group (P=.07).

**Conclusions:**

The fact that the adjunctive access to ASCENSO did not improve outcome could be due to both the rather high effectiveness of TAU and to patients’ limited use of the online platform.

**Trial Registration:**

ClinicalTrials.gov NCT03093467; https://clinicaltrials.gov/ct2/show/NCT03093467

## Introduction

### Background

Depression is one of the most prevalent health problems and the leading cause of disability worldwide [1]. In Chile, the prevalence of this pathology is among the highest internationally. According to data provided by the National Health Survey 2016-2017 conducted by the Chilean Ministry of Health, 15.8% of the general population over 18 reported having experienced depressive symptoms in the past year [2]. Despite the high prevalence of depression and the costs implied for the health systems, a relevant percentage of those suffering from depression do not access treatment. The barriers to accessing effective mental health treatment include individual factors. For example, help-seeking behavior is influenced by the willingness to disclose problems, fear of stigma [3], lack of time for treatment, and sociocultural characteristics. Moreover, barriers regarding providers influence access to treatment, such as scarce screening and diagnose of mood and anxiety disorders within the primary care health setting. Finally, systemic factors, such as the availability of effective treatment, have also been reported to constitute a barrier to accessing mental health treatment [4,5].

The high depression prevalence, its complexity and chronicity [6], and the low rates of treatment access [4] have led to the design and implementation of comprehensive management strategies for the disorder [7-10] including interventions based on information and communication technologies [11].

Internet cognitive therapy (ICT)–based programs often include interactive elements, self-report questionnaires, psycho-education through audio-visual media, and different types of exercises, such as problem solving, recognizing and challenging dysfunctional thinking, activity planning, and behavioral experiments [12,13].

Over the past two decades, many studies have pointed to the effectiveness of ICT-based interventions for the reduction of depressive symptoms [11,14]. Most studies investigated self-management interventions based on internet cognitive behavior therapy (iCBT) with or without clinical guidance that were mostly used as stand-alone interventions (ie, independent of conventional face-to-face treatment). A recent meta-analysis based on 32 studies reported a pooled effect size of *g*=0.67 for the reduction of the depressive symptomatology of iCBT [14]. Nevertheless, most of the included studies addressed self-selected individuals with mild to moderate symptoms and mostly recruited online and outside of clinical settings. It is possible that these individuals differ from those who participate in traditional face-to-face therapies [15], which limits the generalizability of these results.

Another limitation of the available evidence is based on the fact that the majority of trials compared stand-alone iCBT against a weak comparator (ie, wait-list control groups). Furthermore, the comparisons against usual care or active interventions (eg, bibliotherapy) has shown mixed results, some indicating a small [14] or nonsignificant effect [16], whereas others studies conclude that iCBT could be as effective as face-to-face treatment [17-19]. However, there is still a clear lack of high-quality trials addressing this question in clinical samples in which iCBT is tested against evidence-based individual psychotherapy.

In addition to further investigating the potential of such stand-alone interventions, it is considered highly relevant from a service research perspective to investigate to what extent the additional use of ICT-based interventions parallel to conventional face-to-face treatment (ie, blended treatment approaches) may improve depression care. Blended care refers to a combination of online and face-to-face therapy [20]. Blended interventions can have two different objectives: on the one hand, they can aim at increasing the efficiency of treatment by reducing time in the face-to-face setting instead of delivering parts of the treatment via ICT (ie, the assumption is that similar outcomes can be achieved with less face-to-face treatment and therefore at a lower cost) [21]; on the other hand, blended interventions can aim at increasing the effectiveness of treatment by adding adjunctive ICT-based tools to regular face-to-face treatment (ie, the assumption is that the combined treatment will result in improved outcomes) [20,22].

Four recent randomized controlled trials (RCTs) investigated the effectiveness of blended care for depression but showed inconsistent results. Two trials reported statistically significant, medium effect sizes of blended treatment in both inpatient [23] and outpatient settings [24]. These 2 studies included German participants and used the web-based self-help program, Deprexis, for 12 weeks. This program consisted of 10 main modules plus an introductory and a summary module based on cognitive-behavioral techniques, positive psychology, emotion-focused therapy, and dream work. In contrast, 2 other trials found no [25] or only small effects [26] of adding an adjunctive computerized iCBT to face-to-face primary care. Both of these trials included European outpatient samples (UK and German patients) and used the MoodGYM, a web-based CBT program for depression, which consists of 5 interactive modules that are made available sequentially on a weekly basis with a 6-week total duration. Additionally, Gilbody, et al’s [25] 3-group study design allowed for the assessment of an adjunctive iCBT intervention using the Beating the Blues program, which is an interactive, multimedia, computerized CBT package comprising a 15-minute introductory video followed by 8 therapy sessions. The program is entirely online, and there is no interaction with clinicians or individualized feedback on the computer sessions [25]. It is possible that the lack of consistent results among the mentioned studies is due to the diverse samples (inpatients might have more severe symptoms than outpatients) and the different types and durations of the digital intervention program used.

In addition, a large naturalistic study reported that blended interventions for depression and anxiety did not result in improved outcomes and were even associated with longer treatment duration and more costs than was outpatient treatment alone [27].

### Study Objective

In this paper, we report on the first RCT on blended care for depression in Latin America where research on ICT-based interventions is still considered to be in an initial stage [28,29]. In Chile, the first internet-based program for depression, called ASCENSO (based on the acronyms of its Spanish name: Apoyo, Seguimiento y Cuidado de Enfermedades a partir de Sistemas Operativos) was developed and evaluated in a feasibility and acceptance study in 2015 [30].

The main purpose of the present study was to evaluate the effectiveness of a blended treatment approach (ie, ASCENSO offered as adjunctive intervention to depression treatment as usual [TAU] for adult patients) compared to TAU alone.

Based on the high rates of acceptance and satisfaction among patients who actively used the ASCENSO program as well as their recognition of the program as a form of company and a source of help that complements their treatment as reported in the pilot study [30], we hypothesized that the intervention group (IG) would show higher adherence to TAU and a significant decrease in depressive symptoms over time when compared to the control group (CG).

## Methods

### Trial Design

This 2-arm parallel-group RCT compared standard face-to-face psychotherapy plus medication for patients with depression (TAU) with the blended treatment approach, including TAU plus ASCENSO, as a superiority trial. Assessments in both conditions were conducted at baseline (T1), at 6 months (T2), and after 9 months (T3).

### Recruitment, Randomization, and Blinding

Recruitment occurred from May 2017 to February 2019, the study was short 5 participants of the estimated sample size. Recruitment was stopped due to study time lines (once 9-month follow-up assessment was completed) and budget constraints.

Patients seeking treatment for depression at a private, university-affiliated clinical and research mental health care facility in Santiago, Chile, were invited by a research assistant to join the study (offline recruitment).

Inclusion criteria were age between 18 and 64 years, a clinical diagnosis of a current major depressive episode by a staff psychiatrist or mental health–trained physician according to the International Classification of Diseases confirmed by the Spanish Version of the Mini International Neuropsychiatric Interview (MINI) [31], and internet access. Exclusion criteria were a previous suicide attempt (past 10 years); hospitalizations associated with a major depressive disorder (MDD) diagnosis; psychotic episodes; bipolar affective disorder; severe cognitive disability; drug abuse or dependence; antisocial, schizotypal, or borderline personality disorder; serious medical illness; and insufficiency in the Spanish language. Participants were randomized either to the CG receiving psychiatric or psychological TAU or to the IG receiving TAU plus access to ASCENSO. Randomization was balanced, based on a permutated block design, and stratified by MDD episode (first versus 2 or more episodes). The randomization allocation sequence was computer generated and was sent to the research assistant after the participant signed the informed consent. Patients did not receive any incentive or compensation for participating in the study.

Given the design of the study, participants knew to which study group they belonged. In order to avoid potential bias, physicians and psychotherapists were unaware of the group assignment at the beginning of TAU, but they could access this information if participants decided to disclose this information during sessions (for example, by sharing results of the monitoring). Researchers were also unaware of the group assignment (IG vs CG) at the time of analyzing the primary and secondary outcomes of the study. However, when the use of the online platform was being analyzed, this information was transparent.

### Interventions

TAU includes the services typically provided to patients referred by private health insurance companies, within the framework of health difficulties that are covered by Explicit Health Guarantees for Chileans. The benefits are defined by the health authority in a minimum and common package for public and private providers.

The TAU delivered is based on the national clinical guideline for the treatment of depression in individuals age 15 years and older [32]. This indicates the need for nonpharmacological treatment for mild depression and a combination of pharmacological and nonpharmacological treatment for moderate and severe depression. In addition, it indicates follow-up actions for 6 months once the symptomatic decrease or remission is confirmed at 12 weeks of treatment.

In the case of a major depressive episode diagnosis, benefits include monthly care with a mental health–trained primary care physician or a psychiatrist for more severe clinical presentations, access to pharmacological treatment when required, and brief individual psychotherapy.

The usual pharmacological treatment consists of an antidepressant plus anxiolytic, which is prescribed by the physician or psychiatrist according to the severity of the condition, for a minimum period of 6 months. In this sample, 100% (n=167) of patients used antidepressants, 71.9% (120/167) also used anxiolytics, and 55.7% (93/167) used other pharmacological treatment. The psychotherapy considers weekly sessions for 6 to 12 weeks with an extension option upon need.

In more complex cases, the public health or insurance coverage package confers outpatient psychiatric care and short-stay hospitalization or day hospital treatment. At the provider level, regarding patient treatment decisions, benefits defined by the health authority are mostly a reference framework, allowing for clinically based individual adjustments (eg, selection of attending professionals and length and frequency of interventions).

**Figure 1:**
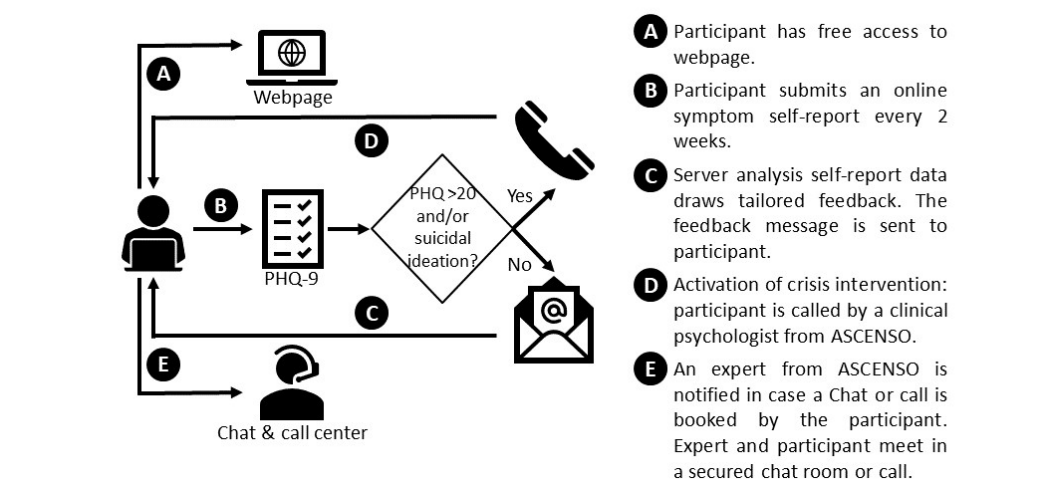
ASCENSO platform functioning. PHQ: Patient Health Questionnaire.

ASCENSO is an online program (see Figure 1 and Multimedia Appendix 1) for monitoring and supporting patients with depression, delivered as adjunctive support to conventional face-to-face treatment. The ASCENSO intervention had a 9-month duration.

ASCENSO’s main aims are delivering psycho-educational information (welcome page, self-care recommendations, and blog components), supportive monitoring and feedback (depressive symptom–monitoring component), and managing emergencies (alarm, online consultation, and emergency information components). Its 7 main components are described as follows: (1) A welcome page that shows the ASCENSO platform objectives is included. (2) Self-care recommendations and psychoeducation involve psycho-educational resources that promote patient self-care and adherence to treatment, specifically, information about the causes, prevention, and treatment of depression. (3) The blog component includes general information about mental health and depression. (4) For the depressive symptom monitoring and feedback component, every 2 weeks, patients report the level of their depressive symptoms by completing the Patient Health Questionnaire-9 online questionnaire [33]. For this purpose, they receive a link to the questionnaire by email (they can also access it via the online ASCENSO platform). If the participant does not respond, a reminder is automatically sent within 5 days by email. The ASCENSO platform evaluates the status of the depressive symptoms with a predefined algorithm and determines whether the patient’s status is improved, unchanged, or deteriorated compared to the previous one. A short feedback message is then sent to the patient (Textbox 1). Each feedback fits the current symptomology and is selected and sent from a database (elaborated by a group of experts). All messages include a brief self-care recommendation, and they promote adherence to psychotherapeutic and psychiatric treatment.

**Examples of feedback messages.**

- We are pleased to know that your current situation is good. We are committed to improving your situation as well. Don't forget to go for a walk or visit someone you like. Do simple things that you like and make you happy.
- We have observed that the last four weeks you have not been well, and that is lamentable news for us. We recommend that you seek a conversation with your therapist or physician. Please note that you can also request a consultation online or by phone in the ASCENSO program. Don't isolate yourself.
- In the questionnaire, you show that your mood has improved a little from your previous results. To continue improving, it is important that you do not forget the self-care recommendations that are available on the ASCENSO program website. Take care of your diet and exercise in moderation, according to your physical possibilities.

(5) An alarm based on symptom monitoring or a “suicide alert” is also included as a component. In the case that monitoring indicates suicidal ideation, the patient automatically receives an email instructing him or her to contact the health center where he or she is being treated and is referred to the “Emergency” section of the menu on the ASCENSO platform. In addition, a professional at the health center responsible for implementing ASCENSO receives an alert notification, evaluates the patient’s situation according to information in the clinical record and symptom report, and acts accordingly.

Regarding the standard operating procedure for suicidality of the trial, the suicide alert was activated by the patient when responses in the ASCENSO platform questionnaire indicated suicide ideation. This alert was automatically sent via email from the platform to a person designated by the health center and the research team; generating 2 different but complementary routes of action. The first was managed by the research team, in which a research assistant (clinical psychologist) contacted the patient within 48 hours after the alert was triggered. It involved verifying the patient's condition, motivating the continuity of the treatment, and remembering the options available on the ASCENSO platform (use of consultation online or by phone). In case the participant could not be contacted, the research assistant made 2 additional attempts in the following days. The second and parallel route of action was the responsibility of the health care center staff. CC reviewed the patient's care and identified if there was a scheduled appointment (either a doctor or a psychologist) during the next 7 days. If the patient did not have an appointment, the health center staff would contact the patient by phone to verify their situation and proceed to follow the internal protocol of the health center for the management of suicidal ideation and suicide attempts.

(6) In the online consultation component, patients can schedule a 30-minute session with a psychologist, which is conducted in a private text-based chatroom or over the phone. Guidance and counseling are provided during the online consultation (sessions do not constitute an online psychotherapeutic process). This component was provided by the mental health center.

(7) For the emergency information component, standard information on what to do and whom to contact in a crisis is provided.

Research assistants registered participants of the IG in the ASCENSO platform and gave them a password to access the intervention. They also offered information on how to access and use the platform. If a participant failed to respond to the first monitoring assessment after 2 weeks, he or she was contacted by phone to clarify potential technical problems. Additionally, during the course of the intervention, the IG participants could contact the research assistants via email when encountering any technical problems on the use of the platform. Participants could access the platform for 9 months.

### Assessment Instruments and Methods 

The MINI Spanish version 6.0.0 was used for assessment in this study. The MINI is a brief and structured interview for major psychiatric disorders of axis I of the Diagnostic and Statistical Manual of Mental Disorders, 4th Edition, and the International Classification of Disease 10. It was specifically designed for implementation in clinical practice and research in psychiatric and primary health care settings and was used in this study at baseline to determine the inclusion and exclusion criteria. The following modules were applied: major depressive episode, suicidality, manic and hypomanic episodes, alcohol and other substance dependence abuse, psychotic and mood disorder with psychotic features, and antisocial disorder. Each module starts with a screening question to exclude the diagnoses and possibly skip the module accordingly if answered negatively or to explore symptoms severity when responded positively. Several validation studies [34] have demonstrated excellent interrater and test reliabilities of the MINI.

The International Personality Disorder Examination (IPDE) Questionnaire and Interview was also used for assessment [35]. The IPDE is a semistructured interview used to assess personality disorders. It has been approved by the World Health Organization and has been translated into Spanish [36]. In this study, it was used to identify exclusion criteria of schizotypal and borderline personality disorders. It also has a brief self-applied screening questionnaire, in which, using dichotomous answers to questions, the individual describes his or her usual behavior in the past 5 years. This questionnaire provides the interviewer with quick information about which personality disorder is likely to be present. The corresponding module of the IPDE interview is then administered, which allows for the confirming or ruling out of the diagnosis.

Demographic information (age, gender, marital status, etc), internet accesses and use, and self-perception of internet expertise were collected through an ad hoc questionnaire.

The primary outcomes were depressive symptoms as assessed by the Chilean adaptation of the Beck Depression Inventory [37]. The Beck Depression Inventory is a 21-item, self-rated scale that evaluates key symptoms of depression; among other items, it includes sadness, pessimism and sense of failure, self-dislike, suicidal ideas, crying, irritability, social withdrawal, indecisiveness, and several somatic preoccupations [38]. Items are scored on a 4-point continuum (0=least, 3=most), with a total score range of 0 to 63. The Chilean adapted inventory has a .92 Cronbach’s α and 1-factor solution [37]. Higher scores indicate greater depressive severity. In this study, the internal consistency was .84, .92,and .94 at basal, 6-month and 9-month assessment, respectively.

Secondary outcomes were quality of life and treatment adherence. Quality of life was measured by the Spanish version of the EuroQol/EQ-5D-3L [39,40]. It includes a short descriptive questionnaire and a visual analogue scale (EQ VAS). The former comprises 5 items: mobility, self-care, usual activities, pain or discomfort, and anxiety or depression. Items are scored on 3 levels: (1) no problems, (2) some problems, or (3) extreme problems. The EQ VAS assessing the respondent’s self-rated health uses a vertical scale where the highest end point is labeled as “The best health you can imagine” (100 points) and the lowest as “The worst health you can imagine.”

Treatment adherence was determined via the Mental Health Center’s internal records. Specifically, we assessed the percentage of treatment sessions attended, including appointments for medical control, psychiatry, and psychotherapy; and percentage of participants who dropped out from TAU (face-to-face interventions).

Finally, for IG participants, the usage of ASCENSO was assessed by server logs.

### Procedures

Regarding the timing when the measurements took place, the MINI, IPDE, and ad hoc questionnaire were applied at baseline measurement, while the Beck Depression Inventory and EuroQol/EQ-5D-3L were used at baseline, 6 months, and 9 months. Information from the health center records and ASCENSO use was collected 9 months after treatment initiation.

As the duration of TAU varied depending on patients’ impairment and their adherence to treatment, fixed assessment intervals were defined as opposed to patients being surveyed at the end of their face-to-face treatment. The assessment at T2 was conducted after 6 months in order to include the recommendation for a minimum time of pharmacological treatment. The assessment at T3 was conducted after 9 months in order to cover the duration of the ASCENSO intervention.

The baseline assessment consisted of web-based self-administered questionnaires that patients completed at a computer provided by the research assistant after they had signed the informed consent. Follow-up assessments consisted of the same web-based self-administered questionnaires but were not completed in the health care center.

### Sample Size

With a medium effect size (*d*=0.5), .05 α error, and .90 of power, G*Power software [41] indicated a sample size of 140 participants (70 in each group). The final sample size established was 172 participants, with a potential attrition of approximately 20% being accounted for.

A medium effect size was assumed, based on the effectiveness of previous eHealth interventions for depression. The reported effect varies from medium-large effect size (0.78) superiority of the computerized CBT over the control group [42] to small-medium (0.41 or 0.34) effect size when internet-based psychological interventions are compared to active control conditions [43,44]. The effect size assumed in our trial was comparable to the effects reported for other blended interventions in 2 recent RCTs [23,24].

A similar approach was used to estimate the sample attrition. Two previous Chilean studies on eHealth technologies reported 10% [45] and 34% [30] sample attrition. In this context, an average estimate of the sample loss was calculated at 20%.

### Analytical and Statistical Approaches

The primary analysis was based on the intention-to-treat principle. A multiple imputation procedure was used to generate 100 multiply imputed data sets, which were further analyzed by using standard procedures for complete data and by combining the results from these analyses [46]. The imputation model considered sex, age, and whether or not the MDD episode was the patient’s first.

Effects on depressive symptoms and quality of life were tested using mixed analysis of variance (based on a mixed models’ approach) to compare changes in assessments from baseline to 6 and 9 months (time factor) between the IG and CG (group × time factor). Additionally, within- and between-group effect sizes were estimated by Cohen’s *d*_z_ and *d*_s_,respectively [47].

Because of their nonnormal distributions, face-to-face treatment adherence indicators were tested with Mann-Whitney tests and chi-square tests (Yates correction). A result was considered statistically significant at P<.05. All analyses were performed using SPSS version 20 (IBM Corp) except for some imputed parameters (P values) which were estimated by R software (“miceadds” package; The R Foundation for Statistical Computing).

### Ethics and Clinical Trial Registration

The Ethical Committee of the Mental Health Center Psicomédica Research Group, Santiago, Chile, approved the study protocol. Clinical trial registration was made under ClinicalTrials.gov (NCT03093467).

## Results

### Participant Characteristics

**Figure 2:**
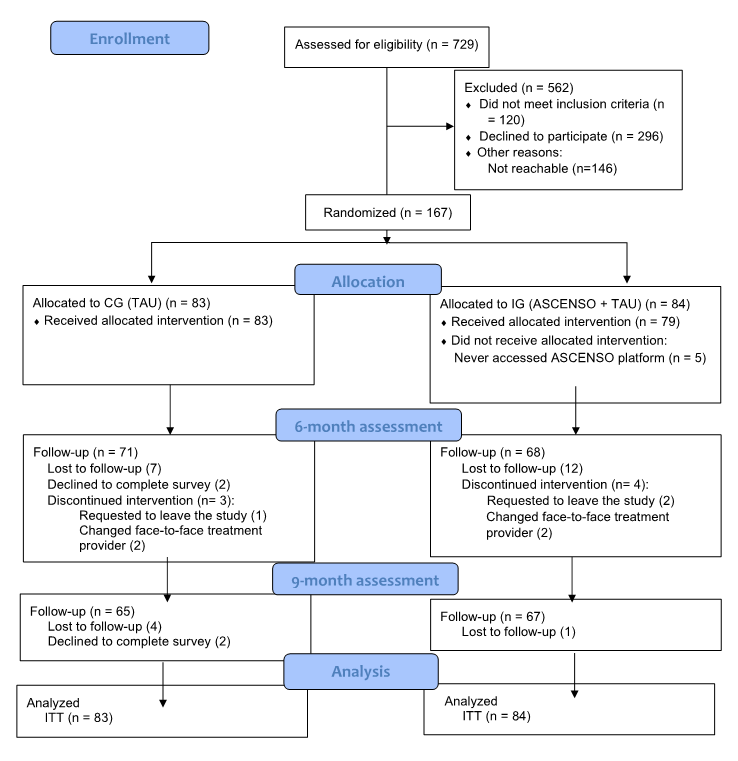
Consolidated Standards of Reporting Trials (CONSORT) diagram (patient flow). CG: control group; IG: intervention group; ITT: intention to treat; TAU: treatment as usual.

Of the 729 patients screened for eligibility, 167 were randomized. The rest either refused to participate, could not be contacted, or did not meet inclusion criteria. Of the 120 patients that did not meet the inclusion criteria; 40% (48/120) had a previous suicide attempt; 18.3% (22/120) did not have confirmed MDD; 12.5% (15/120) had been in treatment for more than 1 month, 8.3% (10/120) had borderline personality, 6.7% (8/120) had a bipolar disorder; 4.2% (5/120) were older than 65 years old; 3.3% (4/120) had no internet access; 1.7% (2/120) had a psychotic episode, and 5% (6/120) were excluded for other reasons. In terms of assessment, 81% (68/84) of the IG and 86% (71/83) of the CG patients completed the 6-month assessment, and 80% (67/84) of the IG and 78% (65/83) of CG patients completed the 9-month assessment (Figure 2).

Of the 167 participants, 76.0% (127/167) were women, 46.1% (77/167) did not have previous MDD episodes, and 82.6% (138/167) were employed. The average age was 39.31 years (range 20-63 years). Most of the participants (49.1%, 82/167) were married or cohabiting and had more than 12 years of education (76%, 127/167). In terms of internet access, 92.8% (155/167) had access at home and 95.2% (159/167) had access through their cell phone; most participants (55.1%, 92/167) self-identified as intermediate internet users. With respect to clinical characteristics, there were no differences between the IC and CG in depressive symptoms or quality of life (Table 1).

Quality of life measured by EQ VAS was associated with a lower probability of missing data at 6 months (odds ratio [OR]=0.97, 95% CI 0.94-0.99; P=.04) and 9 months (OR=0.96, 95% CI 0.94-0.99; *P*=. 01). Sex, age, first MDD episode, marital status (married or cohabiting vs single, divorced, or widowed), educational level, baseline depressive symptoms, and quality of life (measured with EuroQol/EQ-5D-3L) were not associated with the presence of missing data at either measurement interval.

**Table 1:** Participant characteristics at baseline.

Demographic variables			Total (N=167)		CG^a^ (N=83)		IG^b^ (N=84)		Contrast and/or P values^c^	
Age (years), mean (SD)			39.31 (10.84)		39.06 (10.67)		39.56 (11.06)		t_165_=0.30, P=.77	
Age (years), range			20–63		21–62		20–63		N/A^d^	
Female, n (%)			127 (76)		62 (75)		65 (77)		χ^2^_1_= 0.17, P=.69	
First MDD^e^ episode, n (%)			77 (46)		37 (45)		40 (48)		χ^2^_1_=0.16, P=.69	
**Marital status, n (%)**										χ^2^_2_=1.95, P=.38^f^
	Single	58 (35)		27 (33)		31 (37)				
	Married/cohabiting	82 (49)		40 (53)		42 (50)				
	Divorced/separated	24 (14)		15 (18)		9 (11)				
	Widowed	3 (2)		1 (1)		2 (2)				
**Living with children, n (%)**										χ^2^_3_=2.92, P=.40
	No children	79 (47)		36 (43)		43 (51)				
	1 child	42 (25)		21 (25)		21 (25)				
	2 children	35 (21)		18 (22)		17 (20)				
	3 or more children	11 (7)		8 (10)		3 (4)				
**Formal education, n (%)**										P=.45^g^
	Fewer than 8 years	4 (2)		3 (4)		1(1)				
	8-12 years	36 (22)		16 (19)		20 (24)				
	More than 12 years	127 (76)		64 (77)		63 (75)				
**Occupational status, n (%)**										χ^2^_2_=2.33, P=.31^h^
	Housewife	9 (5)		4 (5)		5 (6)				
	Student	11 (7)		3 (4)		8 (10)				
	Worker	138 (83)		70 (84)		68 (81)				
	Unemployed	9 (5)		6 (7)		3 (4)				
**Self-reported internet access and use, n (%)**										
	Internet at work (yes)	111 (67)		55 (66)		56 (67)		P=.99^i^		
	Internet at home (yes)	155 (93)		78 (94)		77 (92)		P=.77^i^		
	Internet by cell phone (yes)	159 (95)		77 (93)		82 (98)		P=.17^i^		
	Checks email ≥2 times a day	118 (70)		51 (61)		67 (80)		P=.01^i^		
**Self-perception of internet expertise, n (%)**										χ^2^_2_=0.07, P=.96
	Basic^j^	33 (20)		17 (21)		16 (19)				
	Intermediate^k^	92 (55)		45 (54)		47 (56)				
	Advanced^l^ or expert^m^	42 (25)		21 (25)		21 (25)				
**Clinical variables, n (%)**										
	Depression Symptoms	26.14 (SD 8.71)		25.00 (SD 8.65)		27.27 (SD 8.67)		t_165_= 1.70, P=.09		
	Quality of life: EuroQol/EQ-5D-3L	8.43 (SD 1.55)		8.25 (SD 1.40)		8.61 (SD 1.68)		t_165_= 1.48, P=.14		
	Quality of life: EQ VAS	50.26 (SD 17.14)		51.34 (SD 17.69)		49.20 (SD 16.61)		t_165_= –0.80, P=.42		

^a^CG: control group (treatment as usual).

^b^IG: intervention group (ASCENSO + treatment as usual).

^c^P values associated to the IG versus CG contrast based on *t* test value and their degrees of freedom (eg, t_165_=1.70), chi-square test values and their degrees of freedom (χ^2^_2_=2.33), or the P values of Fisher’s exact probability test.

^d^N/A: not applicable.

^e^MDD: major depressive disorder.

^f^Collapsed categories: single and widowed.

^g^Fisher’s exact probability test (1-tailed). Collapsed categories: fewer than 8 years and 8–12 years.

^h^Collapsed categories: housewife and unemployed.

^i^Fisher’s exact probability test (2-tailed).

^j^Indicated by a response of “I can turn on my computer, connect to the internet, and send emails”.

^k^Indicated by a response of “I can use some computer programs and find what I want on the internet”.

^l^Indicated by a response of “I can use several computer programs and learn to use a new one”.

^m^Indicated by a response of “I can program a computer, install operating systems, and configure networks”.

### Usage of ASCENSO

Of the 84 participants in the IG, 79 (94%) accessed ASCENSO during the intervention period (9 months); among these, 33 (42%, 33/79) visited the “Welcome” section, 29 (37%, 29/79) visited the “Self-Care Recommendations” section, and 15 (19%, 15/79) visited it multiple times (between 2 and 5 times). The “Emergency Information” section was accessed by 20 participants (25%, 20/79), and 23 participants (29%, 23/79) accessed the online consultation section, but only 6 participants reserved an appointment for a chat (n=3) or telephone (n=3) consultation. Only 5 participants (6%, 5/84) never accessed the online platform.

IG participants answered 49% (785 out of 1595 emails) of the biweekly symptom monitoring questionnaires. Of the platform users (n=79), most participants answered the monitoring questionnaires irregularly. Specifically, 6 participants (8%, 6/79) completed all of the monitoring questionnaires, 24 (30%, 24/79) completed 75% to 99% of the monitoring questionnaires, 14 (18%, 14/79) completed 50%-74%, 8 (10%, 8/79) completed 25%-49%, 26 (33%, 26/79) completed 1%-24%, and only 1 (1%, 1/79) did not answer any symptom questionnaires.

Based on the participants’ responses to monitoring (n=79), a total of 167 suicide alerts were triggered by 51 participants (65%, 51/167): 18 of them (18/51, 35%) triggered it once, 14 (14/51, 27%) triggered it 2 to 3 times, 7 (7/51, 14%) 4 times, and 12 (12/51, 24%) 5 times or more. The participants who triggered alarms reported suicidal ideation on average 3.27 times (SD 2.86, range 1 to 16).

### Primary Outcome

For the primary outcome of depressive symptoms, the mixed-model indicated that group (*F*_1,10689_=1.03; P=.31) and group x time (*F*_2,6087_= 0.48; P=.62) effects were not statistically significant. In contrast, there was a time effect (*F*_2,850_=78.25; P<.001; see Table 2), indicating that depressive symptoms decreased from baseline (mean 26.14; 95% CI 24.57-27.71) to 6 months (mean 14.14; 95% CI 12.38-15.88; P<.001) in both groups, but no such change at 9 months was observed (mean 13.80; 95% CI 12.03-15.57; P=.76). Consistent with previous results, within-group effect sizes were large (*d*= –0.95 to –1.08) at 6- and 9-month assessments in both the CG and IG.

**Table 2:** Imputed means, SDs, and effect sizes for quality of life and depressive symptoms.

Groups		Baseline (T1), mean (SD)	6 months (T2), mean (SD)	9 months (T3), mean (SD)	Within-group effect sizes (*d*_z_)			Between-group effect sizes (*d*_s_)	
					T1-T2	T1-T3	T2^a^		T3^b^
**Depressive Symptoms (BDI-IA^c^)**									
	CG^d^	25.00 (8.65)	13.62 (9.87)	13.52 (11.49)	–1.03	–0.95	–0.10		–0.05
	IG^e^	27.27 (8.67)	14.65 (11.36)	14.07 (11.75)	–0.98	–1.08			
**Quality of life (EuroQol/EQ-5D-3L)**									
	CG	8.25 (1.40)	6.93 (1.80)	6.95 (1.85)	–0.69	–0.63	–0.14		–0.10
	IG	8.61 (1.68)	7.17 (1.63)	7.12 (1.67)	-0.72	–0.65			
**Quality of life (EQ VAS)**									
	CG	51.34 (17.69)	69.16 (19.96)	69.48 (21.34)	0.60	0.76	0.16		0.10
	IG	49.20 (16.61)	65.92 (20.72)	67.39 (21.74)	0.75	0.74			

^a^Between-group effect sizes comparing CG versus IG at time 2 (T2).

^b^Between-group effect sizes comparing CG versus IG at time 3 (T3).

^c^BDI-IA: Beck Depression Inventory-Spanish version.

^d^CG: control group (treatment as usual).

^e^IG: intervention group (ASCENSO + treatment as usual).

### Secondary Outcomes

The secondary outcome of quality of life (EuroQol/EQ-5D-3L) improved in both groups (*F*_2,1067_=37.87; P<.001), but group (*F*_1,9550_=1.64; P=.21) and group x time effects (*F*_2,7678_=0.24; P=.79) were not statistically significant (Table 2). The scores decreased from baseline (mean 8.43; 95% CI 8.18-8.68) to 6 months (mean 7.05; 95% CI 6.76-7.33; P<.001); however, there was no change at 9 months (mean 7.03; 95% CI 6.74-7.33; P=.93). Within-group effect sizes were medium-large (*d*= –0.63 to –0.72) at 6- and 9-month assessments in both the CG and IG.

Similarly, for the EQ VAS (scale from 1 to 100), the model did not indicate statistical significance for either group (*F*_1,11863_=1.23; P=.27) or group x time (*F*_2 6670_=0.13; P=.88) effects; that is, the quality of life did not differ between the groups over time. In addition, there was a time effect (*F*_2,4390_= 51.69; P<.001) in both groups’ quality of life increase from baseline to 6 months (baseline: mean 50.27, 95% CI 47.30-53.24; 6 months: mean 67.54, 95% CI 64.32-70.76; P<.001), but there was no change at the 9-month assessment (mean 68.44; 95% CI 65.13-71.75; P=.91). Again, within-group effects were medium-large size (*d*= –0.60 to –0.76) at 6- and 9-month follow-up in both the CG and IG.

### Treatment Adherence

Table 3 shows that there were no differences between groups (all P values >.05) when considering both the number of appointments and percentage of attended sessions with each type of face-to-face treatment professional: psychiatrist, physician, or psychologist. Across the different components of TAU, the median of participants attended approximately 70% of appointments (except for CG participants at psychiatry sessions).

Additionally, health center reports at the 9 months follow-up indicated that there were no differences between the groups related to dropout rates (P=.07). The dropout rate for face-to-face treatment component was 54% (45/84) for IG versus 39% (32/83) for CG.

**Table 3:** Face-to-face treatment adherence.

Face-to-face intervention rate		IG^a^ values				CG^b^ values				P value^c^	
		Mean (SD)	Median (IQR)	Number	Mean (SD)		Median (IQR)	Number			
**Medical/physician control**											
	Appointments	7.44 (3.87)	8 (6)	82	7.26 (4.19)		7 (6)	81	.53		
	Attendance percentage^d^	71.27 (20.66)	75 (26.16)	82	73.05 (18.50)		71.43 (28.19)	81	.87		
**Psychiatry**											
	Appointments	5 (5.07)	2 (10)	14	6.42 (6.18)		3.50 (10)	26	.48		
	Attendance percentage	51.51 (47.12)	73.90 (100)	14	46.23 (41.54)		63.39 (83.65)	26	.59		
**Psychotherapy**											
	Appointments	14.99 (9.77)	12.50 (14)	84	16.89 (10.14)		13 (14)	83	.15		
	Attendance percentage	74.35 (17.37)	76.33 (24.18)	84	72.64 (15.85)		75.0 (22.67)	83	.39		

^a^IG: intervention group (ASCENSO + treatment as usual).

^b^CG: control group (treatment as usual).

^c^*P*=bilateral significance of the Mann-Whitney test.

^d^Percentage of attendance: number of attended sessions divided by the total number of appointments.

## Discussion

### Principal Findings

The aim of this study is to evaluate the effectiveness of an adjunctive online intervention to enhance face-to-face treatment of patients with depression. This is the first RCT carried out in a Latin American health care setting on this type of blended care, in which part of the treatment was carried out face to face, with other parts being delivered online and with no reduction of any component of the TAU in either the duration or frequency of sessions.

Currently, there is an ongoing discussion about the optimal way to blend internet and face-to-face interventions [18,48-50], with some limited evidence existing regarding the effectiveness of these blended treatments [23-27,51].

As the results indicated, while both groups showed large within-group effect sizes, the blended approach did not improve the effectiveness of the usual care delivered at the mental health center either in terms of reduction of depressive symptoms or in the improvement of quality of life. One possible explanation for this lack of superiority may be the type and intensity of face-to-face treatment and the high effectiveness of TAU.

### Comparison With Prior Work

Previous studies have shown that internet interventions are effective in reducing depressive symptomatology when they are compared to a waiting list and placebo [14,52,53]. In fact, ICT-based interventions compared to a waiting list have shown increased effect compared to those including TAU as a control group [14] but have shown no superiority compared to active control groups [16]. The pre-post effect sizes of TAU and the face-to-face treatment adherence results of our study indicate that this comparator consisting of comprehensive medical and psychological care might have been too strong to leave room for ASCENSO to improve outcomes. In contrast, in the 2 previous RCTs that demonstrated the effectiveness of blended care for depression, TAU was associated only with small or moderate effect sizes [23,24].

A second explanation for the results obtained is related to the heterogeneous use of the ASCENSO platform by patients. In fact, a common problem on internet-delivered psychological treatments is the high degree of nonadherence or dropouts [54,55], which limit their effectiveness. This has also been observed in 2 other RCTs on blended interventions for depression in primary care. Both the studies of Gilbody et al [25] and Löbner et al [26] reported extremely low utilization of their very brief internet-based interventions. Compared to these studies, the uptake and utilization of the online intervention in the our study were higher, with only 6% (5/84) participants never logging in to the program and 52% (44/84) of them completing half or more than half of the supportive monitoring assessments over an intervention period of 9 months.

In fact, some theoretical models have been proposed to account for the nonadherence phenomenon in the eHealth literature. For example, Johansson and colleagues [56] indicated that internet treatment characteristics such as workload, text-content complexity, and a demanding treatment process, which do not match personal prerequisites such as daily routines, language skills, and treatment expectations, respectively, are at the base of this nonadherence phenomenon. Other reasons for nonadherence indicated by the authors were presence of negative effects and lack of face-to-face contact.

Also, regarding the possible causes of the heterogeneous use of the ASCENSO platform, aspects such as the credibility of eHealth interventions should also be taken into account and could be at the base of the differences found in the use among the IG participants. In regard to face-to-face psychotherapy, patient-perceived treatment credibility represents a personal belief about a treatment’s logicalness, suitability, and efficaciousness, which varies in a continuum from negative to positive [57]. Meta-analyses have shown both that positive expectations [58] and the perceived credibility toward the treatment [59] are associated with symptom improvement although their impact is small in magnitude. Given the relative novelty of the use of technologies in the treatment of mental health problems or their use as a complementary tool to usual treatment in local settings, it could be hypothesized that the lack of a user’s personal experience with these technologies (or their lack of presence in a potential user’s social network) could generate negative expectations toward them, and therefore individuals might be less willing to use them.

In comparison to the aforementioned internet treatment characteristics, the ASCENSO platform can be considered a low-demand intervention. Its design is such that it is the patients’ themselves who decide when and how to use the platform since the patients have to access the website and read simple psychoeducational information or actively request assistance through telephone or chat. Only the supportive monitoring goes “from the platform to the end user” and demands the patient’s attention. Therefore, it is the only situation that could be perceived as an overload. However, future research should focus on ways to enhance adherence to ASCENSO and allow us to distinguish when the non-use of this platform indicates that people are using it according to their perceived needs.

Despite there being a dearth of studies in the Latin American context on internet-based interventions in mental health, the few studies conducted tend to show high levels of acceptability but relatively low levels of use and adherence [28,30]. The differential use of technology has been related in some investigations to the levels of patients’ familiarity with technological devices and interventions; that is, their technological literacy [60]. Additionally, the levels of personalization of the interventions and the proximity of the content are important [61].

A subsequent qualitative study that addresses expectations and experiences of patients may help to clarify the reasons for (non)adherence and dropout from the online intervention and would help us to clarify to what extent patients adapt the use of the available eHealth components to their needs. Moreover, this kind of research could inform us how to improve future online and blended interventions along the lines of persuasive design and user-developer co-design tools to favor user adherence [62-66].

Although the appropriate way to measure effectiveness in clinical trials is through intention-to-treat analysis, it accounts for intervention effectiveness regardless of the degree of treatment compliance. However, the effect of offering a program is not necessarily the same thing as the impact of participating on it. Therefore, intention -to-treat analysis does not make “what works for whom” clear [55,67]. Thus, using a “treatment on the treated” analysis that is able to show the impact of program participation could be another future line of research, which would be particularly interesting if used in contexts where it is feasible to encourage more participation.

### Strengths and Limitations

The strengths of this study are its design (an RCT with an active control group), the use of structured interviews to confirm clinical diagnosis, and the measurement of treatment utilization and adherence to TAU via objective records from the health care provider. Although many studies on ICT-based interventions have been criticized for the online and community-based recruitment of participants with mild to moderate symptom severity, participants in this study were recruited in a clinical setting and showed medium to severe depression.

This study also has several limitations including difficulties in the blinding group assignment to patients and medical staff, difficulties in standardizing the measurement time (based on the complex nature of the intervention which contains different interacting components), the use of self-report scales for clinical variables, and the limited availability of adequate indicators to measure the use. These factors limit the generalizability of the results to medium to severe depressive patients with low comorbidities. This is a relevant limitation since the recruitment process showed that a considerable number of individuals had 1 or more comorbidities, which was considered to be an exclusion criterion in this study, particularly a history of previous suicidal ideation or attempts.

Additionally, the fact that the face-to-face treatment was carried out in a private health center could have biased the sample by excluding people with low socioeconomic status and those who lack health insurance (eg, undocumented immigrants). Although the Chilean explicit health guarantee system has expanded the coverage of depression treatment, guaranteeing care, ensuring its quality, and establishing a financial protection system (by defining a maximum copayment), the existence of a payment requirement could be an access barrier for people with fewer economic resources.

Finally, one of the limitations of the current study is its limited replicability since it was implemented in the context of routine health care, which might have been influenced by extraneous variables despite the use of randomization. Nonetheless, it is important to point out that although carrying out a study in a natural context makes replication difficult, doing so provides ecological validity [68], showing the usefulness (or not) of the intervention tool as it would be used in routine health care. In fact, there is extensive research that reports difficulties in replicating the results of manualized or highly standardized interventions in applied contexts, in which results are often less promising [69]. Thus, our findings may be a valuable contribution to decision-makers in routine health care settings.

### Conclusions

This study allows us to progress the understanding of the effectiveness of blended therapy. The study adds to previous studies that have demonstrated that ICT-based interventions can feasibly be implemented in Latin America [28,29,62], a context with high levels of mental health problems [1] and low access to professional treatment [5]. However, the blended treatment approach of ASCENSO did not prove superior in this trial compared to TAU alone. This result has ecological validity since it was implemented in a routine health care setting, offering valuable information for decision-makers about the usefulness of implementing automated systems as a complement to usual care.

At a local level, the results obtained regarding the low use of the platform and lack of improvement in depressive symptoms compared to the effect of only face-to-face treatment are different from what was observed in the European context [70]. These differences might indicate that it is not enough to adapt interventions and that it is increasingly necessary to develop them based on the particular sociocultural, economic, and social health characteristics of the users [61]. Mental health treatment in Latin America is still limited, with scarce specialized human resources, low frequency of treatment sessions, and insufficient postdischarge controls, especially in rural and remote areas. For this reason, it is necessary to continue exploring remote internet-based interventions, especially in Chile where internet penetration is massive [71] and because it has one of the highest prevalences of depressive disorders worldwide [72].

The relevance of the development of effective treatment strategies that reduce or do not require face-to-face contact has increased due to the recent global pandemic we have been facing since the COVID-19 outbreak. It is important to make treatment accessible even in lockdown conditions and respond to the reported associated negative impact this pandemic has had on mental health. This is particularly relevant for the Chilean context where it has been reported that after the COVID-19 outbreak, more than 60% of assessed individuals are experiencing a negative emotional impact and are expressing concerns about the future, general health, work instability, and the current political instability the country [73].

Future interventions might benefit from more actively and explicitly integrating the contents and activities of the ASCENSO platform into the face-to-face therapeutic space. For example, using the results of questionnaires or suicide alarms (among others) as part of the information discussed in the therapeutic session may be useful. Thus, achieving greater integration between the technological and face-to-face treatment components may potentially enhance these components. This would allow patients to process the information provided by the algorithms of the platform, so that they do not feel a mismatch between what the program is offering them and their true state or necessities, a condition that has previously been identified as an important barrier to the use of internet-based Interventions [56,74].

Furthermore, future interventions need to carefully study the motivation and preferences of potential users. In addition, public health efforts to increase awareness about mental health issues and provide guidance on where to seek and receive help seem to be required to ensure that more patients receive appropriate, professional help.

## Appendix

Multimedia Appendix 1 ASCENSO platform overview.

Multimedia Appendix 2 CONSORT eHealth checklist (version 1.6.1).

## Abbreviations

CBT: cognitive behavioral therapy
CG: control group
iCBT: internet-based cognitive behavioral therapy
ICT: internet cognitive therapy
IG: intervention group (IG)
IPDE: The International Personality Disorder Examination
MDD: major depressive disorder
MINI: The Mini International Neuropsychiatric Interview
RCT: randomized controlled trial
TAU: treatment as usual
